# Surface Modified β-Ti-18Mo-6Nb-5Ta (wt%) Alloy for Bone Implant Applications: Composite Characterization and Cytocompatibility Assessment

**DOI:** 10.3390/jfb14020094

**Published:** 2023-02-09

**Authors:** Michael Escobar, Oriol Careta, Nora Fernández Navas, Aleksandra Bartkowska, Ludovico Andrea Alberta, Jordina Fornell, Pau Solsona, Thomas Gemming, Annett Gebert, Elena Ibáñez, Andreu Blanquer, Carme Nogués, Jordi Sort, Eva Pellicer

**Affiliations:** 1 Departament de Física, Universitat Autònoma de Barcelona, 08193 Bellaterra (Cerdanyola del Vallès), Spain; 2 Departament de Biologia Cel lular, Fisiologia i Immunologia, Universitat Autònoma de Barcelona, 08193 Bellaterra (Cerdanyola del Vallès), Spain; 3 Institute for Complex Materials, Leibniz Institut für Festkörper- und Werkstoffforschung Dresden e.V., 01069 Dresden, Germany; 4 Institució Catalana de Recerca i Estudis Avancats (ICREA), Pg. Lluís Companys 23, 08010 Barcelona, Spain

**Keywords:** titanium alloy, electrodeposition, ceramic coating, indentation, corrosion resistance, cell proliferation

## Abstract

Commercially available titanium alloys such as Ti-6Al-4V are established in clinical use as load-bearing bone implant materials. However, concerns about the toxic effects of vanadium and aluminum have prompted the development of Al- and V-free β-Ti alloys. Herein, a new alloy composed of non-toxic elements, namely Ti-18Mo-6Nb-5Ta (wt%), has been fabricated by arc melting. The resulting single β-phase alloy shows improved mechanical properties (Young’s modulus and hardness) and similar corrosion behavior in simulated body fluid when compared with commercial Ti-6Al-4V. To increase the cell proliferation capability of the new biomaterial, the surface of Ti-18Mo-6Nb-5Ta was modified by electrodepositing calcium phosphate (CaP) ceramic layers. Coatings with a Ca/P ratio of 1.47 were obtained at pulse current densities, −j_c_, of 1.8–8.2 mA/cm^2^, followed by 48 h of NaOH post-treatment. The thickness of the coatings has been measured by scanning electron microscopy from an ion beam cut, resulting in an average thickness of about 5 μm. Finally, cytocompatibility and cell adhesion have been evaluated using the osteosarcoma cell line Saos-2, demonstrating good biocompatibility and enhanced cell proliferation on the CaP-modified Ti-18Mo-6Nb-5Ta material compared with the bare alloy, even outperforming their CaP-modified Ti-6-Al-4V counterparts.

## 1. Introduction

For many years, commercially available pure α-titanium (cp-Ti) and its alloys, such as Ti-6Al-4V, have been widely used as bone implant materials. However, Ti-6Al-4V, being a dual-phase α + β alloy, presents a higher modulus of elasticity (125 ± 2 GPa [1]) compared with that of bone (11.4–21.2 GPa [2]). This hinders the transfer of load to the bone, and it produces a stress-shielding effect that can cause implant loosening and may result in premature bone failure [3,4,5]. Additionally, it has been pointed out that the release of V and Al ions can cause hematological and biochemical alterations, osteomalacia, peripheral neuropathy, and Alzheimer’s disease [4,6]. One way to overcome these problems is with the use of β-phase Ti alloys free of V and/or Al. These alloys possess high specific strength, good fatigue resistance, sufficient toughness, excellent corrosion resistance, good formability, and, most importantly, a low elastic modulus in the range of 33–85 GPa [7,8]. Moreover, β-phase stabilizing elements usually confer these alloys excellent biocompatibility [5,9]. Such is the case for Mo, which shows an increased ability to stabilize the β phase [9,10] and low cytotoxicity, being an essential element for the human body [11]. In a similar way, besides not having toxic effects on cells, Nb and Ta are both regarded as biocompatible elements that are able to promote the generation of vascularized, loose connective tissue when in contact with the organism [5]. Nb is also used to further ensure the stabilization of the β phase and improve the alloy’s processability during fabrication, while Ta enhances the mechanical properties and corrosion resistance of the resulting alloy [12]. Thus, it has been demonstrated that Ti alloys containing Mo, Nb, and/or Ta exhibit good biocompatibility and corrosion resistance in human body fluids without causing allergic reactions; at the same time, they have a low elastic modulus and overall superior mechanical properties, which make them suitable for applications as environmentally neutral construction materials and biomedical devices, more specifically orthopedic implants [8,10,13,14].

Unfortunately, the metallic implant surface in its as-cast or standard finished state, e.g., ground or polished with a natural passive layer, usually does not provide sufficient bioactivity. Namely, it is not very efficient in stimulating bone tissue formation and healing, hindering in this way the bone recovery process [15]. To overcome this drawback, the deposition of calcium phosphates (often denoted as CaP for simplicity) onto the implant has been proposed as a strategy to increase the osseointegration [15,16,17]. Different techniques to produce this type of coating are available. Alternatively, dry techniques directly deposit particles onto the substrate without the need of a solvent [15]. Among them, thermal spraying (plasma, flame, and high-velocity oxygen fuel (HVOF) spraying) and physical vapor deposition (PVD) (e.g., magnetron sputtering) can be mentioned. However, these methods either suffer from high production costs or yield coatings with high internal residual stress, low crystallinity, and poor control over the structure of the coating [18]. In addition, some of them operate at temperatures higher than 1000 ºC, which is above the β transus temperature of Ti alloys, thus affecting the alloy microstructure underneath the coating. Alternatively, wet techniques include sol-gel and electrodeposition, which, thanks to their low production costs and high flexibility, are a promising alternative to their dry counterparts [15]. More specifically, electrodeposition can be regarded as a very convenient method to deposit CaP since it provides better control over the coating thickness, the grain size, and the microstructure to a great extent [18,19].

Electrodeposited CaP coatings can be categorized into four main compositions, namely dicalcium phosphate dihydrate (DCPD) or brushite, octacalciumphosphate (OCP), hydroxyapatite (HA), and tricalcium phosphate (TCP) [15]. Of them, HA (Ca_10_(PO_4_)_6_(OH)_2_), with a Ca/P atomic ratio of 1.67, is chemically and structurally similar to the mineral of human hard tissue. Indeed, it exhibits good stability when surrounded by the variable pH and corrosive environment of the body fluid [20,21]. Closely related to it, calcium-deficient hydroxyapatite (Ca_10-x_(PO_4_)_6-x_(HPO_4_)_x_(OH)_2-x_, CDHA) shows higher solubility and better bioresorbable properties, and it can induce the precipitation of bone-like apatite more efficiently than HA [20,21,22,23]. Therefore, by surface-modifying β-phase Ti alloys with CDHA, it should be possible to produce an implant material with improved mechanical properties and biocompatibility.

Most electrolyte formulations for CaP electrodeposition consist of calcium nitrate tetrahydrate (Ca(NO_3_)_2_·4H_2_O) and ammonium dihydrogen phosphate (NH_4_H_2_PO_4_) at pH = 4–5 [24]. At sufficiently large current densities, corresponding to more negative potentials than the water decomposition, the electrodeposition process involves water/proton reduction at the cathode (Equations (1) and (2)), which cause pH value variations at its near-surface regions and, ultimately, the dissociation of dihydrogen phosphate ions via an acid-base reaction (Equations (3) and (4)):

(1) $$2H_{2}O+2e^{-}\to H_{2}↑+2OH^{-}$$

(2) $$2H^{+}+2e^{-}\to H_{2}↑$$

Depending on the pH value rise, then, the resulting hydrogen phosphate ions (HPO_4_^2−^) for pH values between 7.2 and 12.3 (Equation (3)) or the phosphate ions (PO_4_^3−^) for pH > 12.3 (Equation (4)), precipitate together with Ca^2+^ and OH^−^ ions on the cathode, yielding the above-mentioned CaP phases [24]. The eventual precipitation of HA is given as an example in Equation (5):

(3) $$H_{2}PO_{4}^{-}\to HPO_{4}^{2-}+H^{+}$$

(4) $$HPO_{4}^{2-}\to PO_{4}^{3-}+H^{+}$$

(5) $$5Ca^{2+}+3PO_{4}^{3-}+OH^{-}\to Ca_{5}{\left(PO_{4}\right)}_{3}\left(OH\right)$$

If the current densities are lower, then other species different from water (NO_3_^−^ or O_2_) contribute to creating the required scenario for CaP precipitation [19]. Note that although the term “electrodeposition” is frequently utilized in this field, the formation of a coating does not involve the reduction of ions to their zero-valent state as would be the case with metal electrodeposition. Instead, the electroreduction of electrolyte species generates the agents and/or required pH value for subsequent chemical reactions and the precipitation of CaP phases on the electrode.

Although direct current (DC) electrodeposition has been used to coat metallic substrates with CaP layers, pulse current (PC) electrodeposition shows several advantages. Local concentrations of calcium and phosphate ions change under DC electrodeposition, especially for long deposition times, which makes the control of the stoichiometry of the CaP layer (particularly when HA is targeted) difficult. The introduced resting time enables the ions to diffuse and restore the initial concentration of the electrolyte in the whole solution. In addition, the evolved hydrogen gas bubbles under DC become adsorbed onto the surface, giving rise to porosity and worsening the mechanical properties of the coatings. During the resting time between the pulses, hydrogen bubbles can migrate from the working electrode, allowing for a more homogeneous and dense deposition of the CaP [15,18].

In the present work, with the objective of following the research and experimentation on the fabrication of materials for their possible application as bone implants, a new β-phase Ti alloy (Ti-18Mo-6Nb-5Ta (wt%)) was synthesized by arc melting, and the surface of disk-shaped specimens was further coated with CaP-based films by PC electrodeposition. The mechanical properties of both the base alloy and the resulting calcium-deficient HA (CDHA) coatings grown on top were studied by nanoindentation and scratch tests. For the sake of comparison, commercially available Ti-6Al-4V alloy was also coated with CDHA. Finally, cytocompatibility and cell adhesion of the composite biomaterials were evaluated using the Saos-2 cell line, and the results were compared with those for the bare alloys (β-Ti-18Mo-6Nb-5Ta and Ti-6Al-4V). 

## 2. Materials and Methods

### 2.1. Materials Preparation

Commercial Ti-6Al-4V rod (grade 5-ASTM B348) of 3 mm in diameter was purchased from Goodfellow Cambridge Limited. The new β-phase Ti alloy was fabricated by adding the corresponding amounts of titanium (Ti), molybdenum (Mo), niobium (Nb), and tantalum (Ta) to the proposed composition. The pieces of each metal were then placed in a mini arc-melting system (MAM-1) with an arc-melting generator type Lorch Handy TIG 180 DC basic plus pumping system HVT 51/G under high vacuum. The material was melted several times in order to obtain a homogeneous blend, and subsequently, the melted alloy was forced to enter a cold mold by suction so that 4 mm diameter rods could be obtained by suction casting.

Both commercial Ti-6Al-4V and crafted Ti-18Mo-6Nb-5Ta (referred also as TiAlV and TiMoNbTa for simplicity) rods were cut into disks with a thickness of 2.5 mm and ultrasonically rinsed in ethanol and ultrapure water for 5 min each. Then, the disks were embedded in a thermoplastic resin for polishing with SiC papers up to 4000 grit. Once the disks were extracted from the resin, they were again rinsed in ethanol and ultrapure water, as mentioned before.

For the deposition of CaP, electrical contacts were welded to the metallic disks with Sn-Cu wire to be used as working electrodes. The area of the working electrode was limited by wrapping the disks with Teflon tape, so only their polished faces were available for electrodeposition. Then, the disks were immersed in an electrolytic vessel containing 100 mL of a solution consisting of 0.042 M Ca(NO_3_)_2_·4H_2_O and 0.025 M NH_4_H_2_PO_4_ (pH = 4.17 at 24 °C). The electrolytic vessel was equipped with a thermostat jacket that maintained the temperature of the electrolytic bath at 65 °C with a heating circulator, Julabo F12. In the electrolytic cell, the reference electrode employed consisted of a double junction Ag/AgCl with 0.5 M KNO_3_ outer solution and 3 M KCl inner solution, and a platinum wire was used as the counter electrode. For the electrodeposition, a PC method was implemented with varying pulse current densities (j_c_) of −0.3, −1.8, −5, and −8.2 mA/cm^2^, and pulse on and off times of 1 and 2 s, respectively. Although t_on_ and t_off_ values of the order of minutes are frequently applied in the literature [24], shorter times of 0.5–2 s have also been used [25,26,27]. The j_c_ values were selected considering prior literature in the field of CaP electrosynthesis [15,27]. The number of pulses was adjusted accordingly to have similar overall charges passing through the system, namely 506 cycles (−0.3 mA/cm^2^), 167 cycles (−1.8 mA/cm^2^), 34 cycles (−5 mA/cm^2^), and 20 cycles (−8.2 mA/cm^2^). Note that here the concept of charge is different from the ‘deposition charge’ used in metal electrodeposition. Electrodeposition was performed with a potentiostat/galvanostat, Autolab PGSTAT302N. After electrodeposition, some of the samples were subjected to a NaOH post-treatment for 48 h or 72 h at room temperature and dipped several times in ultrapure water to remove the NaOH excess afterwards.

### 2.2. Characterization of Structural, Physical, and Physicochemical Properties

The composition of the TiMoNbTa alloy, as well as the morphology and composition of the CaP coatings, were studied by field-emission scanning electron microscopy (FESEM) on a Zeiss Merlin, which was equipped with energy-dispersive X-ray (EDX) spectroscopy detector. EDX was performed five times throughout the surface of the samples at an applied voltage of 15 kV, and the mean values are reported. Cuts on the CaP-coated alloys were made using the FIB technique (FIB, Helios 5 CX, Thermo Fisher Scientific, Waltham, MA, USA), and SEM (SEM, Helios 5 CX, Thermo Fischer Scientific, Waltham, MA, USA) was used to image the corresponding coating cross section (and to assess its thickness) with an ICE detector at 5 kV acceleration voltage.

X-ray diffraction (XRD), nanoindentation, and electrochemical corrosion studies were performed to study the crystalline phases, mechanical properties, and corrosion behavior, respectively, of the newly synthesized Ti-based alloy and also of the commercial Ti-6Al-4V, for comparison purposes. In the same way, XRD was used to analyze the crystallinity of the CaP-based coatings. XRD patterns were acquired on a Philips X’Pert diffractometer with Cu Kα radiation (λ = 1.5406 Å) in the 2θ range of 10–100° with a step size of 0.026°. Phase identification was performed using the ICDD PDF2 database. Where appropriate, cell parameters, crystal size, and microstrains were determined by Rietveld analysis of the corresponding XRD patterns.

Nanoindentation experiments were carried out on an Anton Paar TriTec Nanoindentation Tester NHT^2^ equipped with a Berkovich pyramidal-shaped diamond tip. A maximum load of 250 mN was applied to a 5 × 5 array on the surface of the sample. The reduced Young’s modulus (E_r_) and hardness (H) were derived from the initial part of the unloading-displacement curve by applying the method of Oliver and Pharr [28]. The adhesion of the CaP coatings to the base alloy was evaluated by means of scratching tests on a Nanoindenter XP from MTS equipped with a Berkovich tip, and the results were compared with those for coatings on commercial Ti-6Al-4V alloy.

For the electrochemical corrosion studies, a disk of 2.5 mm height and 4 mm diameter constructed from the new Ti alloy (TiMoNbTa) was used as working electrode. The disk was embedded in epoxy resin and ground gradually with SiC papers up to 2500 grit, being ultrasonicated in water after each grinding procedure for a few minutes, to finally rinse it with ethanol and pure water and leave it to dry in air. For comparison, a disk of 2.5 mm height and 3 mm diameter constructed from Ti-6Al-4V was employed as a reference material. The electrochemical measurements were then carried out in a double-wall glass cell in a three-electrode configuration. Thus, a large concave platinum net was used as counter electrode, while a saturated calomel electrode (SCE = 0241 V vs. SHE) inserted in a Luggin capillary served as reference electrode. Within the cell, a phosphate-buffered saline (PBS) electrolyte (140 mM NaCl, 10 mM phosphate buffer, and 3 mM KCl; Merck Millipore, Burlington, MA, USA) with pH 7.4 at 25 °C, was maintained at 37 ± 1 °C with the aid of a thermostat coupled to the outer wall of the cell. The whole setup, comprising the cell and the electrodes, was controlled by the Solartron SI 1287 Electrochemical Interface. With this, the corrosion behavior of the TiMoNbTa and the Ti-6Al-4V surfaces were evaluated without any external load in PBS for 2 h, and the open circuit potential (OCP) was determined. Taking into account the final OCP, a potentiodynamic polarization scan was subsequently performed on the working electrode, starting at −50 mV vs. OCP till +2 V vs. SCE, with a scan rate of 0.5 mV/s. Measurements were performed in duplicate.

### 2.3. Cytocompatibility Studies

#### 2.3.1. Cell Culture

Human osteosarcoma-derived Saos-2 cells (ATCC HTB-85) were used for the biocompatibility analyses. The disks (TiAlV, TiAlV/CaP, TiMoNbTa, and TiMoNbTa/CaP) were sterilized with absolute ethanol for 30 min and individually introduced into a 24-well plate. Then, 1 × 10^5^ Saos-2 cells were seeded on top of the disks in each well and cultured in DMEM (ThermoFisher Scientific, Waltham, MA, USA) supplemented with 10% fetal bovine serum (ThermoFisher Scientific) under standard conditions (37 °C and 5% CO_2_).

#### 2.3.2. Cell Proliferation and Cell Viability Analysis

Saos-2 cell proliferation was determined by performing an Alamar Blue cell viability test (Thermo Fisher Scientific, USA) at days 1, 3, and 7 after cell seeding. Briefly, after 24 h of cell seeding, disks were moved to a new plate in order to discard cells growing at the bottom of the wells. Fresh medium with 10% Alamar Blue was added, and cells were incubated for 4 h under standard conditions in the dark. Then, the supernatant was collected, and 200 µL of the solution were transferred to a black-bottom Greiner CELLSTAR^®^ 96-well plate (Sigma-Aldrich, Saint Louis, MO, USA). Supernatant’s fluorescence was measured at 590 nm wavelength after excitation at 560 nm on a Spark multimode microplate reader (Tecan, Männedorf, Switzerland). Fresh medium was added to the cultures, and the assay was repeated after 3 and 7 days. Fluorescence values at days 3 and 7 were normalized to values obtained at day 1. Experiments were performed in triplicate.

Cell viability was investigated using the Live/Dead Viability/Cytotoxicity kit for mammalian cells (Invitrogen, Waltham, MA, USA). To carry out these tests, cells were first incubated on top of the materials for 3 days (to allow them to grow), and then the assay was performed following the manufacturer’s protocol. Images from different regions of the disks were taken using an Olympus IX71 inverted microscope with epifluorescence capability.

#### 2.3.3. Cell Morphology and Cell Adhesion Analysis

For cell morphology analyses, the same samples used for the cell viability assay were processed to be analyzed by SEM. Briefly, cells were washed with 0.1 M Sodium Cacodylate buffer, pH 7.4 (CBS), fixed in 2.5% glutaraldehyde in CBS for 45 min at room temperature (RT), and washed again twice in CBS. Cell dehydration was performed in a series of increasing ethanol concentrations (50, 70, 90, and twice 100%) for 8 min each. Finally, samples were dried using hexamethyldisilazane (HMDS; Electron Microscope Science) for 15 min. Samples were mounted on special stubs and analyzed using a SEM (Zeiss Merlin, Jena, Germany) to observe cell morphology.

Cell adhesion was determined through the analysis of focal contacts by actin filaments and vinculin detection. To perform the immunodetection, cells were washed twice in PBS 72 h after seeding onto the disks and then fixed in 4% paraformaldehyde in PBS for 15 min at RT. After another PBS wash, cells were permeabilized with 0.1% Triton X-100 (Sigma) in PBS for 15 min and blocked for 25 min with 1% bovine serum albumin (BSA; Sigma), and 0.5% Tween 20 (Sigma) in PBS at RT. Samples were then incubated overnight at 4 °C with a mouse anti-vinculin primary antibody (Millipore, MAB3574, Burlington, MA, USA) at 2 µg/mL and washed with blocking buffer. Then, samples were incubated with a mixture of Alexa fluor 594-conjugated phalloidin (Invitrogen), Alexa fluor 488 chicken anti-mouse IgG (Invitrogen), and Hoechst 33258 (Sigma) for 60 min in the dark at RT. Finally, cells were washed in PBS, air dried, and mounted on specific bottom glass dishes (MatTek, Ashland, MA, USA) using ProLong Antifade mounting solution (Life Technologies, Carlsbad, CA, USA). Immunofluorescence evaluation was performed in a confocal laser scanning microscope (Confocal Leica SP5, Leica Microsystems GmbH, Wetzlar, Germany).

#### 2.3.4. Statistical Analysis

All data were quantified with GraphPad Prism 8 (GraphPad Software Inc., San Diego, CA, USA) and are presented as the mean ± standard deviation. The data from cell proliferation assays were statistically compared using one-way analysis of variance (ANOVA) with Tukey-Kramer multiple comparison test. A value of *p* < 0.05 was considered to be significant. Significance is represented in the figures using an alphabetical superscript on top of the columns. Values with different alphabetical superscripts mean that they are significantly different, whereas values with the same alphabetical superscripts are not significantly different.

## 3. Results and Discussion

### 3.1. Fabrication and Characterization of the Ti-18Mo-6Nb-5Ta Alloy

In order to fabricate a potentially non-toxic β-Ti alloy, Mo, Nb, and Ta were selected as alloying elements due to their ability to stabilize the β phase, excellent biocompatibility, and low cytotoxicity [5,9,11]. The proposed composition for the new alloy was established according to the so-called molybdenum equivalency (*MoE*), which is a useful parameter for characterizing the β-phase stability [9]. For the selected alloying elements, the *MoE* is reduced to Equation (6):

(6) $$MoE=1\cdot \left(\mathrm{wt}\%\;Mo\right)+0.28\cdot \left(\mathrm{wt}\%\;Nb\right)+0.22\cdot \left(\mathrm{wt}\%\;Ta\right)$$

Here, Mo is used as a reference, and the constant before the concentration of each element represents the ratio between the minimum concentration of Mo and the corresponding element to stabilize the β phase. The previous relation was optimized to obtain a *MoE* above 10 and, in this way, ensure the stabilization of the β phase. Thus, the final nominal composition was Ti-16Mo-5Nb-4Ta (wt%).

**Figure 1:**
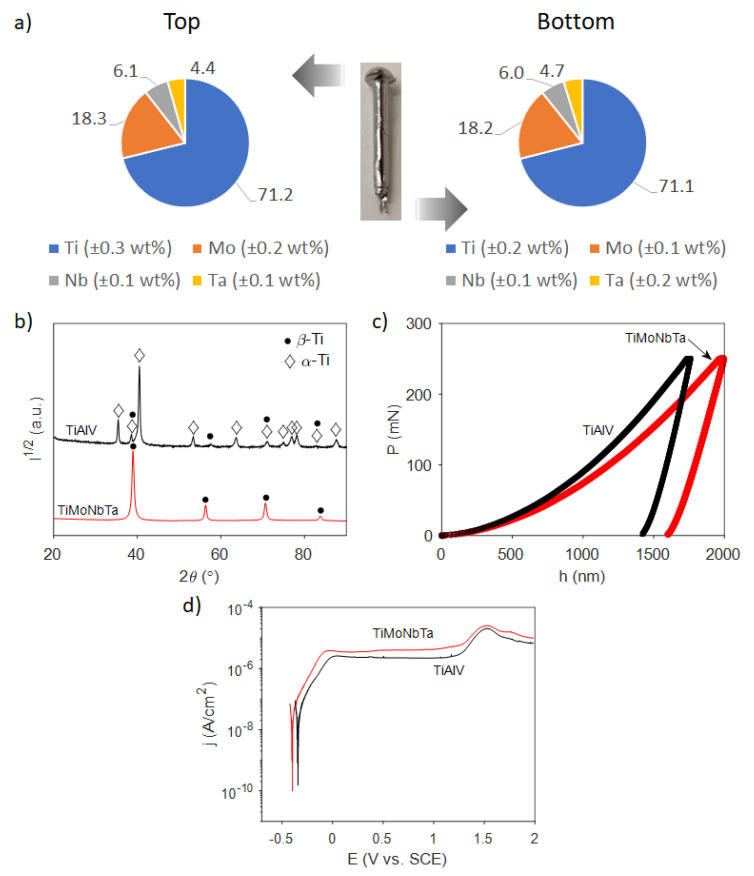
(**a**) Pie charts of the elemental content in wt% at the bottom and top parts of the casted TiMoNbTa rod. (**b**) XRD patterns of TiAlV base alloy (black curve) and TiMoNbTa (red curve). (**c**) Load-unload nanoindentation curves for TiMoNbTa and commercial TiAlV alloys. (**d**) Potentiodynamic polarization curves for TiMoNbTa and TiAlV in PBS solution at 37.5 °C.

After the corresponding amounts of each element were placed in the arc-melter and a rod of the alloy was obtained by Cu mold suction casting, pieces from the top and bottom of it were cut and analyzed with EDX spectroscopy to confirm the homogeneity of the rod. As shown in Figure 1a, the weight distributions of the elements between the bottom and top parts of the rod are very similar, and they correspond to a Ti-18Mo-6Nb-5Ta (wt%) formulation that is close to the nominal one (Ti-16Mo-5Nb-4Ta). Similarly, several disks (of about 1 mm thickness) were cut from the rod and were subjected to XRD. A representative XRD pattern is shown in Figure 1b, together with the XRD pattern of commercial Ti-6Al-4V. The identified diffraction peaks of TiMoNbTa match well with the β-Ti (bcc) phase, which confirms the successful fabrication of an alloy with the desired crystal structure. No other diffraction peaks were observed, contrary to Ti-6Al-4V, where a mixture of α and β phases was found.

Further, the mechanical properties of the new alloy (as well as those of the commercial Ti-6Al-4V) were measured by nanoindentation. Representative curves of the loading and unloading processes recorded during nanoindentation are shown in Figure 1c. Both the reduced Young’s modulus (E_r_) and hardness (H) values were determined from the obtained data and compared between each other and with those reported in the literature for bone (see Table 1).

It can be seen that the new alloy has a considerable hardness (3.4 GPa), albeit one that is lower than that of commercial Ti-6Al-4V (4.2 GPa). The hardness for commercial Ti-6Al-4V is in agreement with the values reported in the literature, namely, 3.6–5.0 GPa, depending on the batch and experimental conditions [1,29]. More importantly, the new alloy also possesses a significantly lower reduced Young’s modulus (79 GPa) than Ti-6Al-4V (106.1 GPa), showing a reduction of almost 25% in this property. This is expected from the lower E_r_ of the β-Ti phase compared with the α-Ti phase. The new elastic modulus is thus closer to the range in which this property varies for the bone. This feature is highly beneficial since it reduces the stress shielding effect caused by a marked difference between the modulus of the bone and the implant, preventing the development of symptoms related to osteoporosis, detachment, and failure of implants, and avoiding revision surgeries [30]. Interestingly, the H/E_r_ ratio (plasticity index), which is taken as an indirect estimate of the wear resistance of a material [31], is slightly higher for the new alloy. This means that the Ti-18Mo-6Nb-5Ta alloy would offer good resistance to wear and abrasion when subjected to repeated cyclic loads or strains, thereby ensuring its long-term success as an implant [32]. The proxy H^3^/E_r_^2^ represents the resistance to plastic deformation, and, as a first approximation, a material with a larger H^3^/E_r_^2^ is less likely to be plastically deformed and should therefore have higher toughness. According to the values gathered in Table 1, the Ti-18Mo-6Nb-5Ta alloy has slightly higher toughness than commercial Ti-6Al-4V. Finally, the energy ratios are similar for both alloys, in particular the U_Pl_/U_Tot_, suggesting that they are able to withstand a similar amount of plastic deformation.

Figure 1d shows the potentiodynamic polarization curves of both the new Ti alloy and commercial Ti-6Al-4V in PBS at 37 °C. Both alloys offer similar corrosion resistance in PBS, yielding close corrosion potential values (−0.341 V and −0.297 V vs. SCE for Ti-6Al-4V and Ti-18Mo-6Nb-5Ta, respectively). Corrosion current densities are well below 1 μA/cm^2^, revealing very low free corrosion and metal ion release. In turn, passive current densities are in the range of 2.2–4.0 μA/cm^2^. Therefore, it can be concluded that the corrosion resistance of Ti-18Mo-6Nb-5Ta is similar to that of the widely established Ti-6Al-4V material.

### 3.2. Electrodeposition of CaP Coatings and Their Characterization

**Figure 2:**
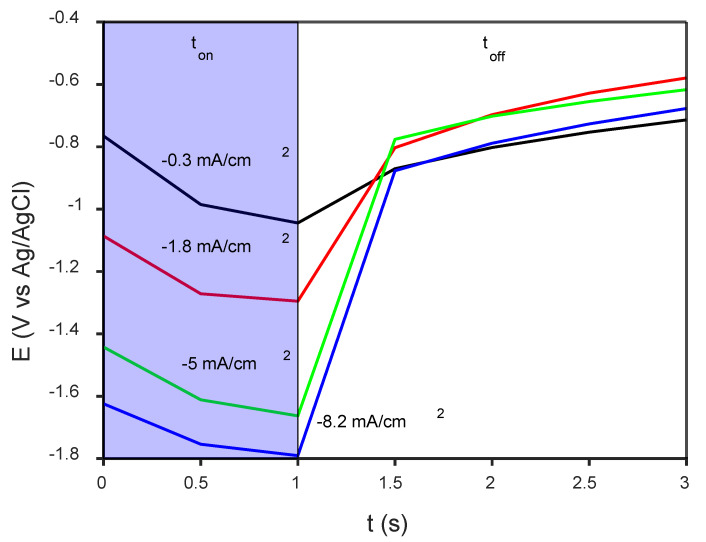
E-t transient recorded during last cycle at the indicated PC densities. Data acquisition was performed in steps of 0.5 s. t_on_ and t_off_ times applied were 1 and 2 s, respectively, and the overall charge (Q) was kept constant for every deposition (0.5 C/cm^2^). The electrolyte solution (100 mL) contained 0.042 M Ca(NO_3_)_2_·4H_2_O and 0.025 M NH_4_H_2_PO_4_.

Once the base alloy was successfully produced, disk-shaped specimens were coated with CaP by PC deposition. For comparison purposes, the Ti-6Al-4V was also coated with CaP under the same experimental conditions. Figure 2 shows the detail of a potential-time (E-t) transient recorded during the deposition of CaP onto the Ti-18Mo-6Nb-5Ta base alloy. During the t_on_, the potential (E) shifts towards more negative values, while E relaxes and approaches the open circuit potential during t_off_. Notice that E at t_on_ = 1 s varies between −1.0 and −1.8 V vs. Ag/AgCl, and hence, reduction of water at values more negative than −1.5 V most likely occurs in our case. According to Eliaz [33], having achieved some nucleation already at −842 mV vs. SCE, potentials below −1.26 V vs. SCE were required to promote HA growth on Ti-6Al-4V substrate when potentiostatic deposition from an electrolyte containing 0.61 mM Ca(NO_3_)_2_ and 0.36 mM NH_4_H_2_PO_4_ at pH = 6 was attempted. Although it is not strictly comparable with a galvanostatic pulse deposition scheme, potentiostatic (current-time) transients recorded during the DC deposition of HA on a glassy carbon electrode were associated with the nucleation and growth of deposits on a conducting surface [34], wherein the current density shifted towards more negative values as the applied potential was made more negative.

**Figure 3:**
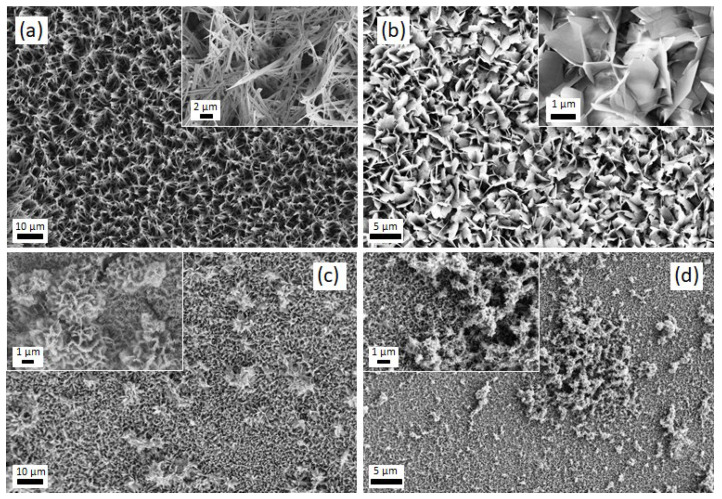
SEM images of CaP coatings deposited on the TiMoNbTa alloy by PC with Q = 0.5 C/cm^2^ and (**a**) j_c_ = −0.3 mA/cm^2^, (**b**) j_c_ = −1.8 mA/cm^2^, (**c**) j_c_ = −5 mA/cm^2^, and (**d**) j_c_ = −8.2 mA/cm^2^. t_on_ and t_off_ times applied were 1 and 2 s, respectively. The electrolyte solution (100 mL) contained 0.042 M Ca(NO_3_)_2_·4H_2_O and 0.025 M NH_4_H_2_PO_4_.

**Figure 4:**
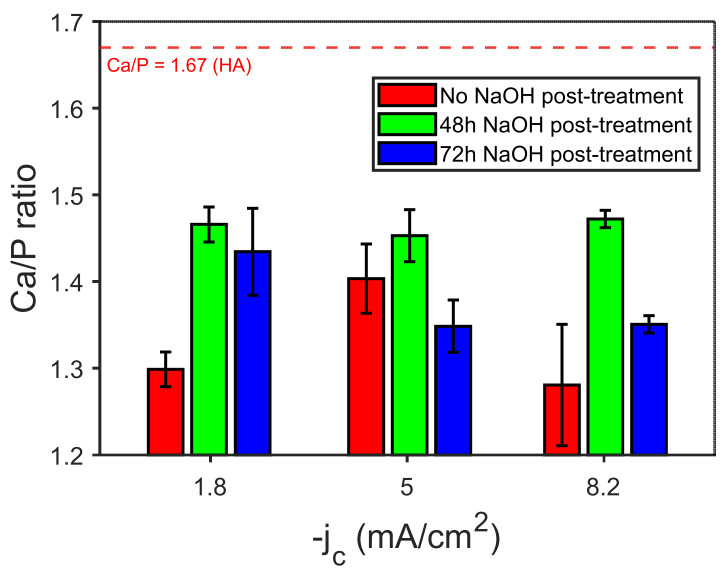
Ca/P atomic ratio in the as-prepared (no NaOH post-treatment) and NaOH-treated coatings produced at different j_c_ values and similar overall charge (−Q = 0.5–0.8 C/cm^2^). The electrolyte solution (100 mL) contained 0.042 M Ca(NO_3_)_2_·4H_2_O and 0.025 M NH_4_H_2_PO_4_.

**Figure 5:**
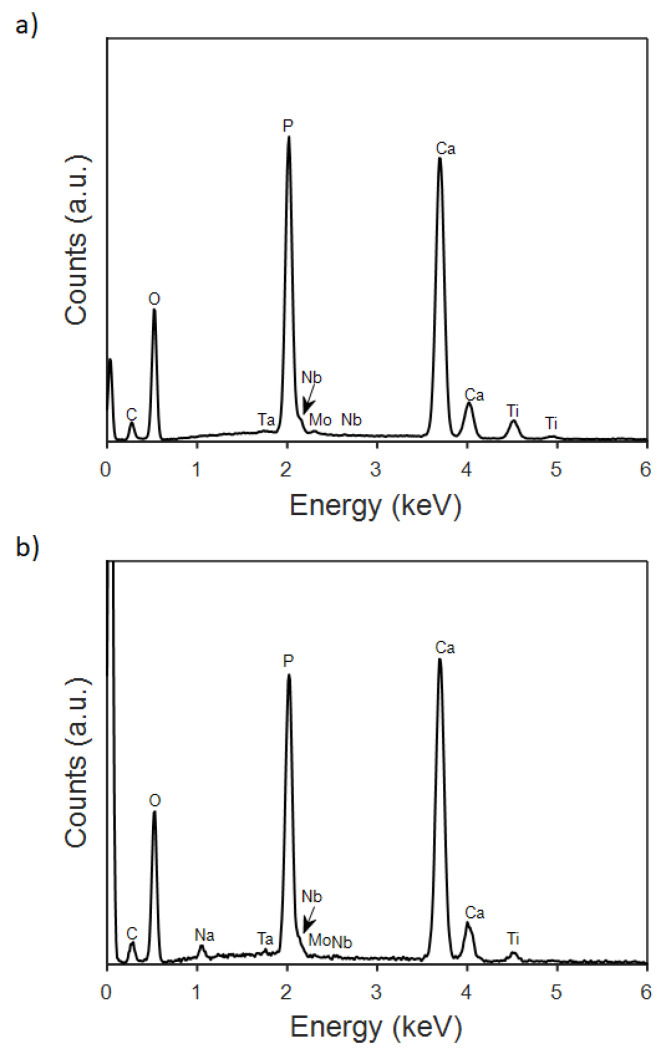
EDX spectra of CaP coatings deposited on the TiMoNbTa alloy by PC at j_c_ = −1.8 mA/cm^2^, t_on_ = 1 s, t_off_ = 2 s, and Q = 0.8 C/cm^2^ in (**a**) as-deposited state and (**b**) after immersion in 0.1 M NaOH for 48 h.

Figure 3 shows the SEM images of the CaP coatings obtained at varying j_c_ values. The morphology of the coatings changed from belt- to flake-like as j_c_ was made more negative, i.e., −0.3, −1.8, and −5 mA/cm^2^ (Figure 3a–c). The insets show magnified details of the coating morphology, in which the formation of a belt network or grass-like morphology is appreciated. In parallel, the Ca/P ratio increased from 1.2 to 1.3. Further increase of the current density to −8.2 mA/cm^2^ caused the precipitation of oxyhydroxides, likely due to local variation of the pH value caused by intensified hydrogen coevolution, without bringing about a notorious increase in the Ca/P ratio. The mean Ca/P ratios varied between 1.28 and 1.40 (Figure 4). A representative EDX spectrum of an as-deposited CaP coating is given in Figure 5a. The pattern shows that both the element peaks originated from the coating layer and the alloy underneath. Considering that the spectra had enough counts and that the Ca and P peaks are isolated, the minimum detectable mass for elements with Z > 11 can ideally be as low as 0.02% wt. In practice, the detection limit of EDX in modern SEMs is about 1–2% wt. for light atoms, as it is in the case of calcium and phosphorous [35,36]. Hence, the estimated Ca/P ratios must be taken with caution, although the trends are meaningful.

A powerful strategy that has been considered to promote the formation of HA is an alkaline post-treatment [15], e.g., a post-synthesis immersion in NaOH solution. Since the growth of HA requires an excess of OH^−^ ions, such alkaline treatment will supply the sufficient OH^−^ concentration, which in combination with Ca^2+^ and $PO_{4}^{3-}$ will result in the transformation of intermediate CaP phases into HA [15]. The Ca/P ratios determined by EDX of coatings that were immersed in NaOH 0.1 M for different time periods (48 and 72 h) after electrodeposition at varying current densities are summarized in Figure 4.

**Figure 6:**
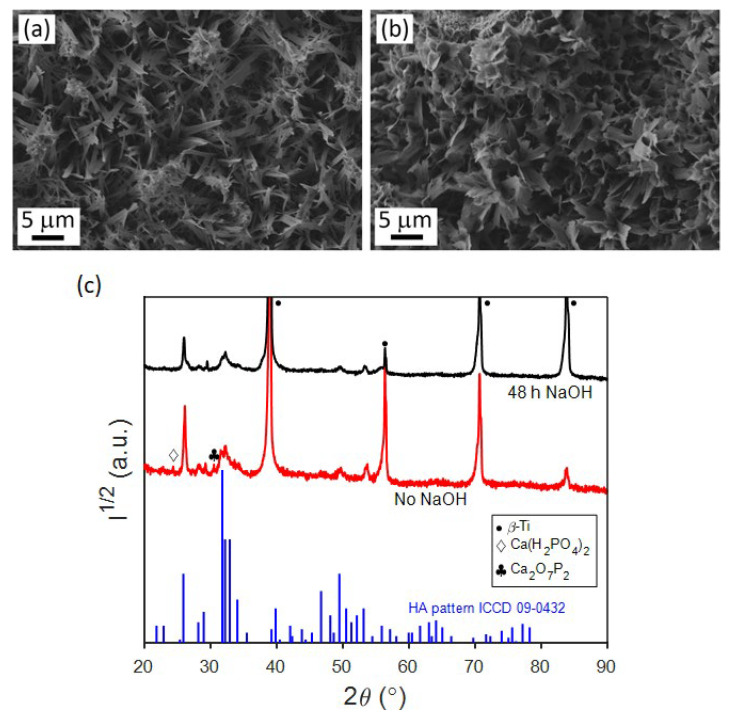
SEM images of CaP coatings deposited on the TiMoNbTa alloy by PC at j_c_ = −1.8 mA/cm^2^, t_on_ = 1 s, t_off_ = 2 s, and Q = 0.8 C/cm^2^ in (**a**) as-deposited state and (**b**) after immersion in 0.1 M NaOH for 48 h, and (**c**) corresponding XRD patterns. The theoretical pattern for HA is shown in blue at the bottom.

It is evident that the post-treatment with NaOH can successfully increase the Ca/P ratio of the as-deposited samples (see Figure 4 and Figure 5). However, higher values of the Ca/P ratio are obtained with 48 h of immersion, while after 72 h this ratio tends to decrease. According to several authors, the increase observed at 48h could be explained by the release of Ca^2+^ and $PO_{4}^{3-}$ ions from the as-deposited coatings when they are soaked in the alkaline solution. These ions would combine with the OH^−^ ions present in solution to yield the HA phase [10]. The decline in the Ca/P ratio at higher immersion times suggests a redeposition of the Ca^2+^ and $PO_{4}^{3-}$ onto the coating. For the samples grown at j_c_ of −1.8 and −8.2 mA/cm^2^ values, the Ca/P ratio can be as high as ~1.47. Notice the relative increase in the peak intensity for Ca, which in turn will increase the Ca/P ratio in Figure 5b. These values match those of CDHA, whose Ca/P can range, according to the literature, from 1.33 up to 1.67 [20]. The CDHA is characterized by higher solubility and consequently more bioactivity than the HA phase [20,37], and it has improved bioresorbable abilities, which stimulate a strong interaction and attachment to the bone thanks to a quick resorption [21]. The increase in Ca/P ratio upon alkalinization treatment for 48 h is accompanied by a change in the on-top morphology of the coatings (Figure 6a,b). Namely, the leaves become wider and adopt a more flower-like structure.

The crystal structure of these coatings was studied by XRD (Figure 6c). The patterns show characteristic peaks for the base alloy and less intense peaks ascribed to the CaP coatings. Although the differences in the patterns for the coatings were quite subtle, on the sample without NaOH post-treatment, the peaks could be attributed to a mixture of different CaP phases: CDHA, monocalcium phosphate monohydrate (Ca(H_2_PO_4_)_2_), and calcium diphosphate (Ca_2_O_7_P_2_). Meanwhile, all the peaks in the pattern of the NaOH-treated coating could be attributed to the CDHA phase. By performing a Rietveld analysis of the XRD pattern of the NaOH-treated coating, the cell parameters, crystal size, and microstrains of both the base alloy and the CDHA coating were determined and are summarized in Table 2.

The cell parameter for the base alloy is *a* = 3.2632 Å and it is microcrystalline (crystal size well beyond 200 nm). Meanwhile, the CDHA coating is nanocrystalline, and the microstrains are rather low considering the deposition method used. In some reports [38] on stoichiometric hydroxyapatite, *a* has been determined to be 9.18 Å, which is lower than the values obtained from the Rietveld fitting of the experimental patterns. This difference might be explained by the deficiency of Ca ions in the hexagonal unit cell [38].

**Figure 7:**
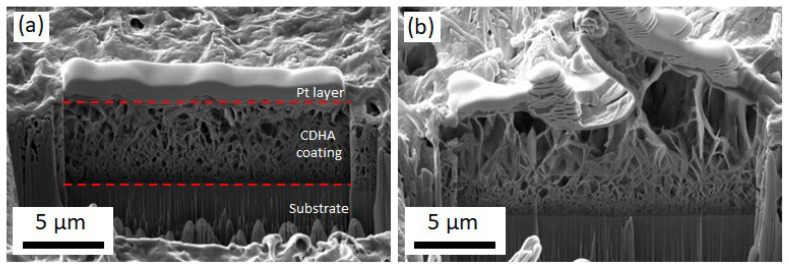
FIB cuts of CDHA coatings grown at j_c_ = −1.8 mA/cm^2^, t_on_ = 1 s, t_off_ = 2 s, and Q = 0.8 C/cm^2^, followed by an NaOH treatment for 48 h, on (**a**) Ti-18Mo-6Nb-5Ta and (**b**) Ti-6Al-4V alloys.

In order to determine the thickness of the CDHA coatings on the Ti alloys, specimens were cut using a FIB. The corresponding SEM pictures are shown in Figure 7, where the average thickness is about 5 μm. In the cross-section SEM images, the overall porous structure of the CDHA can be observed, with pore sizes that vary along depth, from a few nm close to the substrate to μm-sized pores at the upper parts of the films, starting at the interface with the alloy with a more compact morphology and evolving toward a more open structure with larger plate-like features [25]. Note that the flaky morphology observed by SEM in Figure 6b corresponds to the top part of the cross-section view in Figure 7.

**Figure 8:**
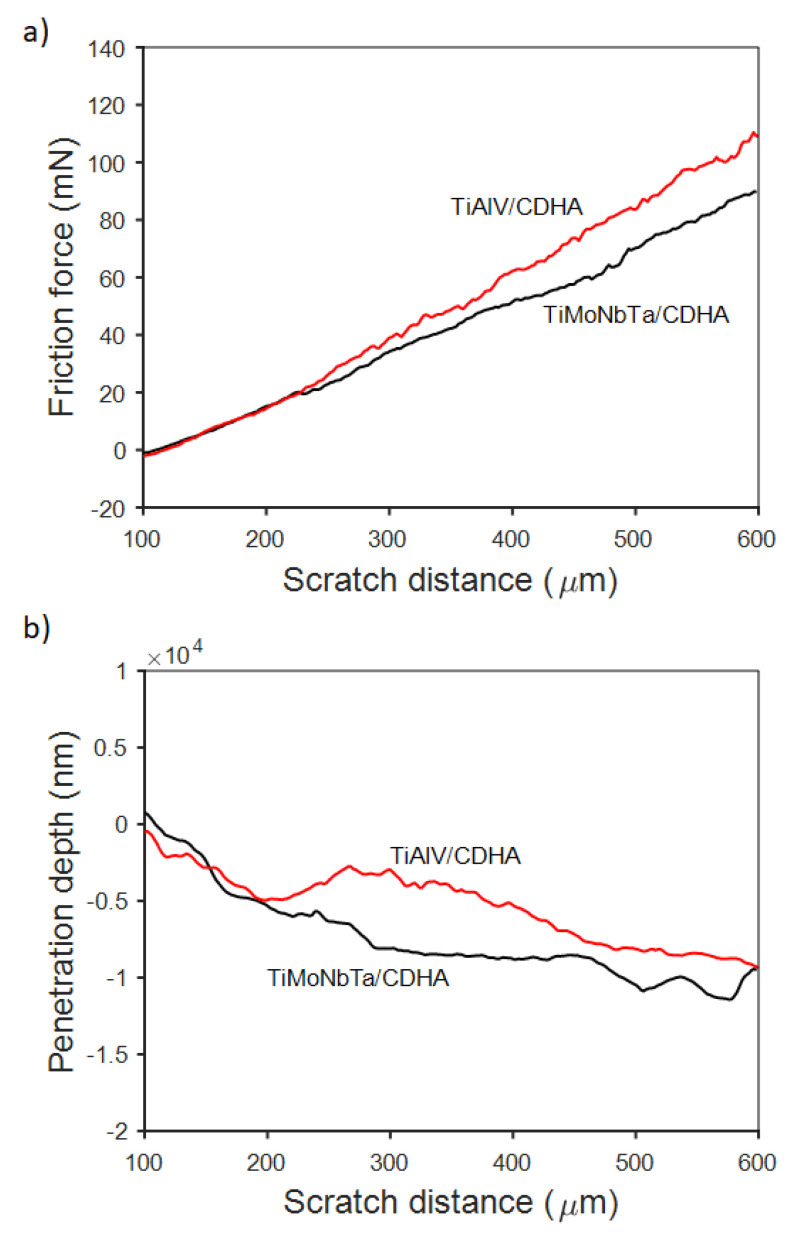
Representative (**a**) friction force and (**b**) penetration depth vs. scratch distance for the CDHA-coated Ti-18Mo-6Nb-5Ta and Ti-6Al-4V alloys using the optimized conditions of Figure 7.

Scratch tests were also performed on the Ti-6Al-4V and the new alloy, both coated with CDHA, as shown in Figure 8. First, the friction force scales linearly with the scratch distance (Figure 8a). The slightly lower frictional forces for the CDHA-coated TiMoNbTa material are likely not significant considering that the deposited CDHA is similar from the morphological and structural standpoints. Importantly, no critical load was determined to cause adhesive failure of the coatings within the explored force range. The penetration depth increases with the scratch distance and reaches 5 μm at approximately 200 μm of scratch distance (Figure 8b). Further recorded penetration depths of around 8 μm might suggest local failure. Hence, the behavior of the coating on the new alloy upon loading follows quite the same trend as that on the commercially used Ti-6Al-4V, which indicates a similar adhesion of the CDHA to the substrate.

### 3.3. Cytocompatibility of CaP-Coated and Uncoated Alloys

The cytocompatibility of the materials was assessed by cell viability, cell proliferation, cell morphology, and cell adhesion analyses. Cell viability analysis determines if direct cell contact with the alloys produces a toxic effect (i.e., a decrease in the number of live cells), whereas cell proliferation analysis allows to assess whether cells cultured on top of the alloys can grow (i.e., increase in number over time).

**Figure 9:**
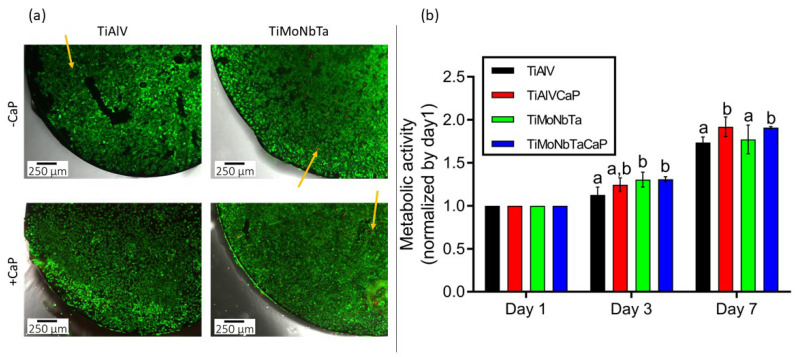
Cytocompatibility of TiAlV, TiAlV/CaP, TiMoNbTa, and TiMoNbTa/CaP alloys. (**a**) Cytotoxicity measured on Saos-2 cells after 3 days in culture using the live/dead kit. Live and dead cells appear in green and red, respectively. Yellow arrows point to dead cells. (**b**) Proliferation of Saos-2 cells grown at 1, 3, and 7 days in culture. Results were normalized by day 1. Different superscripts on top of the columns denote significant differences (*p* < 0.05) among the materials at the same time point.

A Live/Dead kit was used to determine the cytotoxicity of four different samples: TiAlV and TiMoNbTa substrates, plus the previously optimized coated ones, namely, TiAlV/CaP (where “CaP” refers to the CDHA coating) and TiMoNbTa/CaP (where “CaP” also refers to the CDHA coating). As shown in Figure 9a, none of the materials proved to be toxic, as a high number of live cells were observed growing on all the disks’ surfaces after 3 days in culture. Although no quantitative analyses were performed, images revealed a slightly higher number of cells on top of the TiAlV/CaP and TiMoNbTa/CaP alloys when compared with their counterparts without CaP. Furthermore, in all samples, only a few cells were stained red, meaning that none of the materials is cytotoxic.

The proliferation of Saos-2 cells cultured on the different samples was assessed on days 1, 3, and 7 after seeding. Results of the metabolic activity were normalized with respect to the values for day 1 and compared among materials at each time point. As shown in Figure 9b, the proliferation of cells growing on TiMoNbTa (without and with CaP) alloys was higher than that of cells growing on TiAlV at day 3. Furthermore, after 7 days, CaP coating of both TiAlV and TiMoNbTa resulted in higher cell proliferation when compared with alloys without CaP coating. The results of the Alamar Blue seem to be in agreement with the live/dead kit results, confirming that the new TiMoNbTa alloy is good in terms of cell proliferation.

Ti-6Al-4V alloys have long been used as standard bone implant materials because of their excellent reputation for corrosion resistance and biocompatibility. Nonetheless, the long-term performance of these alloys has raised some concerns due to the release of aluminum and vanadium from the alloy [39]. The results here presented are in agreement with those of other authors who have shown that Ti-6Al-4V exhibits good biocompatibility [40]. Furthermore, our results show that the newly developed Ti-18Mo-6Nb-5Ta allows increased cell proliferation while avoiding the use of aluminum and vanadium.

**Figure 10:**
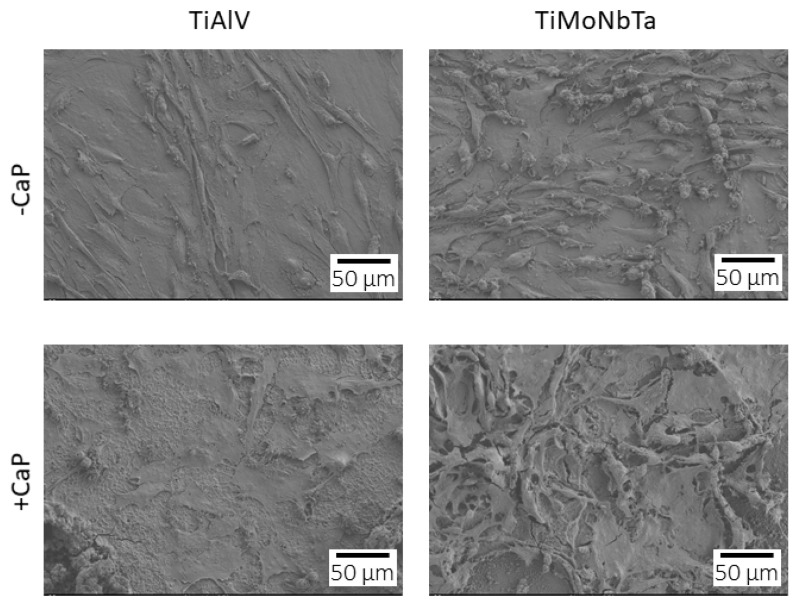
Scanning electron microscope images of human Saos-2 cells cultured for 3 days on TiAlV, TiAlV/CaP, TiMoNbTa, and TiMoNbTa/CaP alloys.

Alternatively, SEM analysis of osteoblasts grown on the different samples showed that cells were randomly distributed on their surfaces after 3 days of culture (Figure 10). Regarding cell morphology, we observed that on all the alloys, cells presented a flattened polygonal morphology with cytoplasmic extensions in different directions. Thus, differences in wettability between uncoated and CaP-coated alloy surfaces do not appear to interfere with cell adhesion.

**Figure 11:**
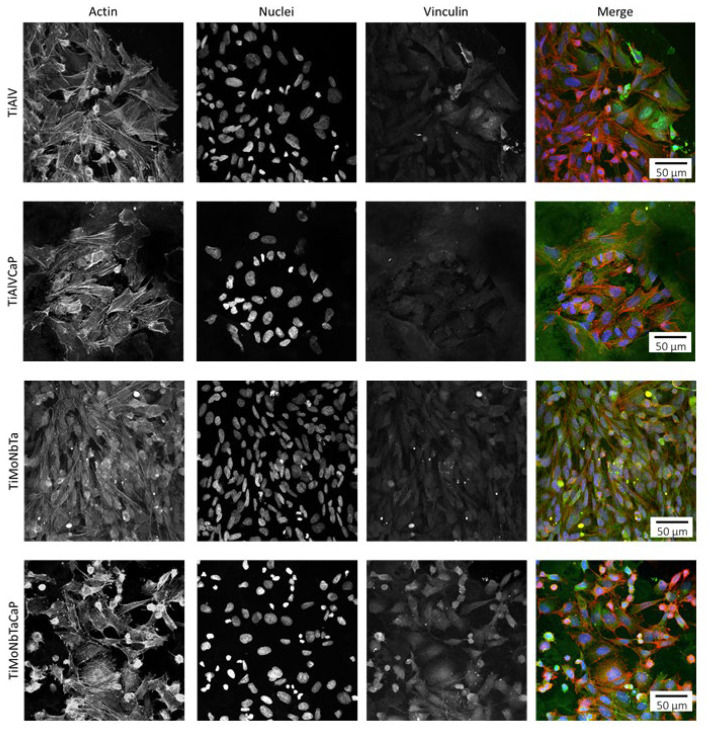
Saos-2 cells adhered to the surfaces of the TiAlV, TiAlV/CaP, TiMoNbTa, and TiMoNbTa/CaP after 3 days in culture. Stress fibers (actin; red), vinculin (green), and nuclei (DNA; blue) can be observed.

Cell adhesion to the alloys was confirmed through immunofluorescence analysis of vinculin and stress fibers (actin). These analyses showed that Saos-2 cells were completely adhered to the samples’ surfaces after 3 days of culture (Figure 11), corroborating the results obtained by SEM, and were able to establish focal contacts. Cells presented well-defined stress fibers, crossing the totality of the cell’s cytoplasm and ending in focal contacts. Most of the stress fibers were found in parallel, and some of them were found without a defined orientation.

According to the results obtained in the cell proliferation, viability, morphology, and adhesion analyses, both TiAlV and TiMoNbTa alloys, with and without CaP coating, are biocompatible. As reviewed by Kolli et al. [9], β-Ti derived alloys present good mechanical and biocompatibility properties, which, according to our results, are both true for TiAlV and TiMoNbTa alloys.

In addition, the surface coating with CaP provides better results over both alloys in terms of cell proliferation when compared with the non-coated ones. CaP has been demonstrated to be a good biocoating for enhancing bone growth in osteogenic materials [41,42], and in the present work, we show that both alloys could benefit from the addition of a CaP in the CDHA structure later at the surface. Furthermore, our results suggest that the use of TiMoNbTa/CaP would provide better osseointegration than TiAlV/CaP.

## 4. Conclusions

A new β-Ti alloy (Ti-18Mo-6Nb-5Ta (wt%)) with non-toxic alloying elements was successfully fabricated by arc melting, using the *MoE* as a predictive tool to obtain the desired β-phase. The resulting alloy showed improved mechanical properties, maintaining a high hardness (3.4 GPa) and lowering its reduced Young’s modulus (79 GPa) by almost 25% in comparison with the value for the commonly used Ti-6Al-4V (106 GPa). This, in combination with the biological advantages provided by the replacement of Al and V with Mo, Nb, and Ta as documented in literature, pointed out the need for fabrication of a superior β-Ti alloy. Simultaneously, the corrosion behavior of PBS was similar to that of Ti-6Al-4V. The potential integration of the alloy into the bone was further enhanced by the functionalization of its surface with a CaP coating produced by electrodeposition. Structural characterization (SEM, EDX, XRD, FIB, and scratching tests) confirmed the formation of a nanocrystalline CDHA coating with a plate/flower-like morphology, a Ca/P ratio of 1.47, a thickness of 5 μm, and good adhesion to the substrate. Finally, the cytocompatibility of the new alloy, uncoated and coated with CDHA, was also assessed by means of cell viability, cell proliferation, cell morphology, and cell adhesion analyses. The results indicated that the bare alloy and the composite material, i.e., TiMoNbTa/CDHA, do not produce a toxic effect since cells attach well to their surfaces, forming anchor points across the area with good morphology. Additionally, the proliferation analysis showed that the growth rate of cells is higher when cultured on the newly fabricated TiMoNbTa alloy than on the commercial TiAlV, and this rate increased further when the material was coated with CDHA, suggesting a higher capacity to promote bone regeneration and growth.

## Figures and Tables

**Table 1:** Reduced Young’s modulus, hardness, and H/E_r_, H^3^/E_r_^2^, U_El_/U_Tot_ and U_Pl_/U_Tot_ ratios experimentally determined by nanoindentation for the newly synthesized Ti-18Mo-6Nb-5Ta and commercial Ti-6Al-4V alloys. Bibliographic data for diaphyseal femoral bone are also given for the sake of comparison.

	Bone [2]	Ti-18Mo-6Nb-5Ta	Ti-6Al-4V
E_r_ (GPa)	11.4–21.2	79.0 ± 3.0	106.0 ± 1.0
H (GPa)	0.23–0.76	3.40 ± 0.10	4.20 ± 0.1
H/E_r_	0.02–0.036	0.044 ± 0.002	0.040 ± 0.001
H^3^/E_r_^2^ (GPa)	0.0001–0.001	0.0070 ± 0.0010	0.0068 ± 0.0005
U_El_/U_Tot_	--	0.231 ± 0.010	0.233 ± 0.004
U_Pl_/U_Tot_	--	0.77 ± 0.02	0.77 ± 0.01

**Table 2:** Cell parameters, crystal size, and microstrains determined by Rietveld fitting of the XRD pattern of NaOH-treated coating deposited on the TiMoNbTa substrate shown in Figure 6c.

Material	*a* (Å)	*c* (Å)	Crystal Size (nm)	Microstrains
Ti-18Mo-6Nb-5Ta (β phase)	3.2632	--	>200	5.3·10^−4^
CDHA (hcp phase)	9.2911	6.8540	16	11.8·10^−4^

## Data Availability

Not applicable.

## References

[1] Tuninetti V., Jaramillo A.F., Riu G., Rojas-Ulloa C., Znaidi A., Medina C., Mateo A.M., Roa J.J. (2021). Experimental Correlation of Mechanical Properties of the Ti-6Al-4V Alloy at Different Length Scales. Metals.

[2] Zysset P.K., Edward Guo X., Edward Hoffler C., Moore K.E., Goldstein S.A. (1999). Elastic Modulus and Hardness of Cortical and Trabecular Bone Lamellae Measured by Nanoindentation in the Human Femur. J. Biomech..

[3] Trincă L.C., Mareci D., Solcan C., Fântânariu M., Burtan L., Hriţcu L., Chiruţă C., Fernández-Mérida L., Rodríguez-Raposo R., Santana J.J. (2020). New Ti-6Al-2Nb-2Ta-1Mo Alloy as Implant Biomaterial: In Vitro Corrosion and in Vivo Osseointegration Evaluations. Mater. Chem. Phys..

[4] Verma R.P. (2020). Titanium Based Biomaterial for Bone Implants: A Mini Review. Mater. Today Proc..

[5] Kuroda D., Niinomi M., Morinaga M., Kato Y., Yashiro T. (1998). Design and Mechanical Properties of New i Type Titanium Alloys for Implant Materials. Mater. Sci. Eng. A.

[6] Xu D., Wang T., Wang S., Jiang Y., Wang Y., Chen Y., Bi Z., Geng S. (2020). Antibacterial Effect of the Controlled Nanoscale Precipitates Obtained by Different Heat Treatment Schemes with a Ti-Based Nanomaterial, Ti–7.5Mo–5Cu Alloy. ACS Appl. Bio Mater..

[7] Guo S., Meng Q., Zhao X., Wei Q., Xu H. (2015). Design and Fabrication of a Metastable β-Type Titanium Alloy with Ultralow Elastic Modulus and High Strength. Sci. Rep..

[8] Mohammed M.T., Khan Z.A., Siddiquee A.N. (2014). Beta Titanium Alloys: The Lowest Elastic Modulus for Biomedical Applications: A Review. Int. J. Chem. Nucl. Metall. Mater. Eng..

[9] Kolli R., Devaraj A. (2018). A Review of Metastable Beta Titanium Alloys. Metals.

[10] Pinotti V.E., Plaine A.H., Romero da Silva M., Bolfarini C. (2021). Influence of Oxygen Addition and Aging on the Microstructure and Mechanical Properties of a β-Ti-29Nb–13Ta–4Mo Alloy. Mater. Sci. Eng. A.

[11] Barceloux D.G., Barceloux D. (1999). Molybdenum. J. Toxicol. Clin. Toxicol..

[12] Majumdar P., Singh S.B., Chakraborty M. (2008). Elastic Modulus of Biomedical Titanium Alloys by Nano-Indentation and Ultrasonic Techniques—A Comparative Study. Mater. Sci. Eng. A.

[13] Sarimov R.M., Glinushkin A.P., Sevostyanov M.A., Konushkin S.V., Serov D.A., Astashev M.E., Lednev V.N., Yanykin D.V., Sibirev A.V., Smirnov A.A. (2022). Ti-20Nb-10Ta-5Zr Is Biosafe Alloy for Building of Ecofriendly Greenhouse Framework of New Generation. Metals.

[14] Gudkov S.V., Simakin A.V., Konushkin S.V., Ivannikov A.Y., Nasakina E.O., Shatova L.A., Kolmakov A.G., Sevostyanov M.A. (2020). Preparation, Structural and Microstructural Characterization of Ti–30Nb–10Ta–5Zr Alloy for Biomedical Applications. J. Mater. Res. Technol..

[15] Safavi M.S., Walsh F.C., Surmeneva M.A., Surmenev R.A., Khalil-Allafi J. (2021). Electrodeposited Hydroxyapatite-Based Biocoatings: Recent Progress and Future Challenges. Coatings.

[16] Picard Q., Olivier F., Delpeux S., Chancolon J., Warmont F., Bonnamy S. (2018). Development and Characterization of Biomimetic Carbonated Calcium-Deficient Hydroxyapatite Deposited on Carbon Fiber Scaffold. J. Carbon Res..

[17] Dumelie N., Benhayoune H., Richard D., Laurent-Maquin D., Balossier G. (2008). In Vitro Precipitation of Electrodeposited Calcium-Deficient Hydroxyapatite Coatings on Ti6Al4V Substrate. Mater. Charact..

[18] Li T.-T., Ling L., Lin M.-C., Peng H.-K., Ren H.-T., Lou C.-W., Lin J.-H. (2020). Recent Advances in Multifunctional Hydroxyapatite Coating by Electrochemical Deposition. J. Mater. Sci..

[19] Schmidt R., Gebert A., Schumacher M., Hoffmann V., Voss A., Pilz S., Uhlemann M., Lode A., Gelinsky M. (2020). Electrodeposition of Sr-Substituted Hydroxyapatite on Low Modulus Beta-Type Ti-45Nb and Effect on in Vitro Sr Release and Cell Response. Mater. Sci. Eng. C.

[20] Beaufils S., Rouillon T., Millet P., Le Bideau J., Weiss P., Chopart J.-P., Daltin A.-L. (2019). Synthesis of Calcium-Deficient Hydroxyapatite Nanowires and Nanotubes Performed by Template-Assisted Electrodeposition. Mater. Sci. Eng. C.

[21] Gecim G., Dönmez S., Erkoc E. (2021). Calcium Deficient Hydroxyapatite by Precipitation: Continuous Process by Vortex Reactor and Semi-Batch Synthesis. Ceram. Int..

[22] Wang L., Wang M., Li M., Shen Z., Wang Y., Shao Y., Zhu Y. (2018). Trace Fluorine Substituted Calcium Deficient Hydroxyapatite with Excellent Osteoblastic Activity and Antibacterial Ability. CrystEngComm.

[23] Zhang H., Zhang M. (2011). Characterization and Thermal Behavior of Calcium Deficient Hydroxyapatite Whiskers with Various Ca/P Ratios. Mater. Chem. Phys..

[24] Drevet R., Benhayoune H. (2022). Electrodeposition of Calcium Phosphate Coatings on Metallic Substrates for Bone Implant Applications: A Review. Coatings.

[25] Mokabber T., Lu L.Q., van Rijn P., Vakis A.I., Pei Y.T. (2018). Crystal Growth Mechanism of Calcium Phosphate Coatings on Titanium by Electrochemical Deposition. Surf. Coat. Technol..

[26] Fornell J., Feng Y.P., Pellicer E., Suriñach S., Baró M.D., Sort J. (2017). Mechanical Behaviour of Brushite and Hydroxyapatite Coatings Electrodeposited on Newly Developed FeMnSiPd Alloys. J. Alloys Compd..

[27] Vidal E., Buxadera-Palomero J., Pierre C., Manero J.M., Ginebra M.-P., Cazalbou S., Combes C., Rupérez E., Rodríguez D. (2019). Single-Step Pulsed Electrodeposition of Calcium Phosphate Coatings on Titanium for Drug Delivery. Surf. Coat. Technol..

[28] Oliver W.C., Pharr G.M. (1992). An Improved Technique for Determining Hardness and Elastic Modulus Using Load and Displacement Sensing Indentation Experiments. J. Mater. Res..

[29] Hynowska A., Blanquer A., Pellicer E., Fornell J., Suriñach S., Baró M., González S., Ibáñez E., Barrios L., Nogués C. (2013). Novel Ti–Zr–Hf–Fe Nanostructured Alloy for Biomedical Applications. Materials.

[30] Campos-Quirós A., Cubero-Sesín J.M., Edalati K. (2020). Synthesis of Nanostructured Biomaterials by High-Pressure Torsion: Effect of Niobium Content on Microstructure and Mechanical Properties of Ti-Nb Alloys. Mater. Sci. Eng. A.

[31] Leyland A., Matthews A. (2000). On the Significance of the H/E Ratio in Wear Control: A Nanocomposite Coating Approach to Optimised Tribological Behaviour. Wear.

[32] Geetha M., Singh A.K., Asokamani R., Gogia A.K. (2009). Ti Based Biomaterials, the Ultimate Choice for Orthopaedic Implants—A Review. Prog. Mater. Sci..

[33] Eliaz N., Eliyahu M. (2007). Electrochemical Processes of Nucleation and Growth of Hydroxyapatite on Titanium Supported by Real-Time Electrochemical Atomic Force Microscopy. J. Biomed. Mater. Res..

[34] Vladislavić N., Rončević I.Š., Buzuk M., Buljac M., Drventić I. (2021). Electrochemical/Chemical Synthesis of Hydroxyapatite on Glassy Carbon Electrode for Electroanalytical Determination of Cysteine. J. Solid State Electrochem..

[35] Falcone R., Sommariva G., Verità M. (2006). WDXRF, EPMA and SEM/EDX Quantitative Chemical Analyses of Small Glass Samples. Microchim. Acta.

[36] Sensitivity/Detection Limit of EDS. https://www.globalsino.com/EM/page4792.html.

[37] Drevet R., Velard F., Potiron S., Laurent-Maquin D., Benhayoune H. (2011). In Vitro Dissolution and Corrosion Study of Calcium Phosphate Coatings Elaborated by Pulsed Electrodeposition Current on Ti6Al4V Substrate. J. Mater. Sci. Mater. Med..

[38] Victor S.P., Kumar T.S.S. (2008). Tailoring Calcium-Deficient Hydroxyapatite Nanocarriers for Enhanced Release of Antibiotics. J. Biomed. Nanotechnol..

[39] Rao S., Ushida T., Tateishi T., Asao S. (1996). Effect of Ti, AI, and V Ions on the Relative Growth Rate of Fibroblasts (L929) and Osteoblasts (MC3T3-El) Cells. Biomed. Mater. Eng..

[40] Navarro M., Michiardi A., Castaño O., Planell J.A. (2008). Biomaterials in Orthopaedics. J. R. Soc. Interface.

[41] Cuijpers V.M.J.I., Alghamdi H.S., Van Dijk N.W.M., Jaroszewicz J., Walboomers X.F., Jansen J.A. (2015). Osteogenesis Around CaP-Coated Titanium Implants Visualized Using 3D Histology and Micro-Computed Tomography. J. Biomed. Mater. Res. Part A.

[42] Surmenev R.A., Surmeneva M.A., Ivanova A.A. (2014). Significance of Calcium Phosphate Coatings for the Enhancement of New Bone Osteogenesis—A Review. Acta Biomater..

